# The Duality of Ray-Based and Pinhole-Camera Modeling and 3D Measurement Improvements Using the Ray-Based Model

**DOI:** 10.3390/s22197540

**Published:** 2022-10-05

**Authors:** Christian Bräuer-Burchardt, Roland Ramm, Peter Kühmstedt, Gunther Notni

**Affiliations:** 1 Department Imaging and Sensing, Fraunhofer Institute for Applied Optics and Precision Engineering IOF, Albert-Einstein-Str. 7, D-07745 Jena, Germany; 2 Machine Engineering Faculty, Technical University Ilmenau, Ehrenbergstraße 29, D-98693 Ilmenau, Germany

**Keywords:** camera modeling, machine vision, camera calibration, photogrammetric stereo sensors, 3D measurements, error analysis

## Abstract

Geometrical camera modeling is the precondition for 3D-reconstruction tasks using photogrammetric sensor systems. The purpose of this study is to describe an approach for possible accuracy improvements by using the ray-based-camera model. The relations between the common pinhole and the generally valid ray-based-camera model are shown. A new approach to the implementation and calibration of the ray-based-camera model is introduced. Using a simple laboratory setup consisting of two cameras and a projector, experimental measurements were performed. The experiments and results showed the possibility of easily transforming the common pinhole model into a ray-based model and of performing calibration using the ray-based model. These initial results show the model’s potential for considerable accuracy improvements, especially for sensor systems using wide-angle lenses or with deep 3D measurements. This study presents several approaches for further improvements to and the practical usage of high-precision optical 3D measurements.

## 1. Introduction

The pinhole-camera model is the most common camera model for 3D-reconstruction tasks using stereo-camera arrangements. Typically, this model, including distortion correction, adequately describes geometric situations and provides very small systematic measurement errors in its 3D-reconstruction results. However, certain conditions may lead to larger systematic measurement errors, such as the usage of fish-eye lenses, extreme-wide-angle lenses, or large measurement volumes with deep measurements.

The achievable measurement accuracy of photogrammetric optical sensors working under such conditions is considerably lower than those of high-end devices. However, there is considerable improvement potential if such sensors use a more specific camera model instead of the pinhole model. Specifically, the rarely used ray-based or generic-camera model may lead to more accurate 3D-measurement results. Ray-based-camera models have been introduced in the literature [1,2,3,4,5,6,7,8,9,10,11], but so far, none have been established for practical 3D dense-reconstruction applications.

The lack of a standard methodology for the calibration process can be considered the most important reason for the low applicability of ray-based-camera models. Although several authors give suggestions for the calibration of measurement systems using the ray-based-camera model, their results are not sufficiently convincing to cause a general change in the generation of suitable software tools. In the next section, an overview of the existing approaches is given.

In this paper, we present a new calibration methodology for the ray-based-camera model and analyze the influence of this model on 3D measurements in comparison to classical pinhole modeling. The main difference between the two camera models is the inclusion in the pinhole model of a common viewpoint, called the projection center, where all rays converge.

## 2. Related Work

Sturm et al. [12] give an extensive description of camera models as the foundation for geometric computer vision. The first description of the ray-based model as a “two-plane model” was given by Martins et al. [1]. The major merit of their work is that it was the first suggestion of the ray-based model in a very simple but effective form. Their error analysis, however, was limited to the determination of the back-projection error. Different interpolation strategies were compared with pinhole calibration, improvements were partly marginal. The influence on 3D reconstruction results was not analyzed. This model was extended by Gremban et al. [2] in 1988. Their main contributions were the complete formulation of the projection problem by a set of linear equations and the suggestion of an appropriate calibration strategy. Champleboux et al. [3] introduced the “N-Planes B-Spline” (NPBS) model, which also means an extension of the two-planes model. They reported that using the NPBS method, an accuracy in the same order of magnitude as the sensor resolution was obtained. The ray-based model is also known as the “generic camera model” [4]. Grossberg and Nayar [5,6] call it the “raxel” model, according to the intention of one-to-one correspondence between pixels and rays. Yu and McMillan describe the general case of cameras with linear ray propagation as general linear cameras and extend this modeling by new multi-perspective camera models [7].

Further research concerning ray-based-camera modeling and calibration has been published by Dunne et al. [8], Sun et al. [9], Pak [10], and Khomutenko et al. [11], among others. Dunne et al. [8] proposed improvements to the standard generic calibration method for central cameras. Sun et al. [9] used the ray-based-camera representation for a new pose-estimation method. Khomutenko et al. [11] introduced an enhanced unified camera model for distortion removal and 3D reconstruction using a single wide-angle or fish-eye camera and showed how epipolar geometry can be applied using this model.

Calibration methods for this kind of camera model have been proposed by several authors. Martins et al. [1] proposed function estimation using a few observation points on a grid at the distance of the two certain model planes. The calibration technique proposed by Gremban et al. [2] was based on finding local linear solutions of the projection problem. Champleboux et al. [3] suggest for the calibration process the estimation of a function from R^2^ to R^4^ using observed data points on two certain planes and smooth bicubic B-spline functions. Sturm and Ramalingam proposed a non-central bundle-adjustment procedure for the calibration of generic cameras [4]. Grossberg and Nayar presented a calibration method using structured light patterns for the extraction of the raxel parameters of arbitrary imaging systems [5,6]. Pak used his new methodology for the calibration of a catadioptric imaging system [10].

Li et al. [13] introduced a calibration methodology for a projection unit which is used as an inverse camera for 3D reconstruction.

The evaluation of the calibration quality is typically performed through the presentation of the global back-projection error. All authors report a decrease in the standard deviation of the error value. However, this quantification does not always give an idea of the achievable accuracy improvement in 3D measurements using stereo-camera arrangements. Wong et al. [14] report a reduction in the probing error and flatness error according to [15].

In this paper, we present a new calibration methodology for the ray-based-camera model and analyze the influence of the model on 3D measurements in comparison to classical pinhole modeling.

## 3. Materials and Methods

### 3.1. Camera Modeling

#### 3.1.1. Pinhole Model

**Figure 1:**
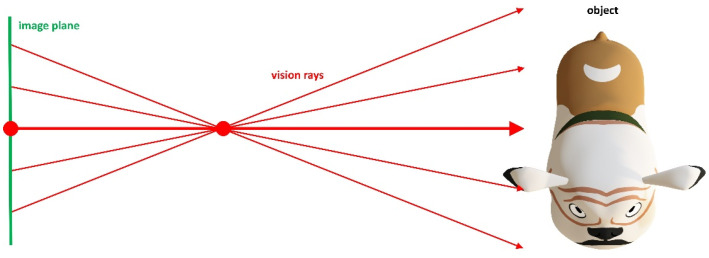
Pinhole-camera model. All rays intersect in a single viewpoint.

The most common camera model used for 3D reconstruction tasks is the pinhole-camera model (PM). It describes the camera geometrically as an image plane and a certain 3D point *O*, called the projection center, in the 3D world co-ordinate system WCS (see Figure 1). All rays from the observed scene pass through *O* before intersecting with the image plane. The intersection points in the image plane are assigned to the discrete picture elements (pixels) of the camera chip. The perpendicular distance of the projection center to the image plane is called principal distance or camera constant *c*. In order to compensate for deviations between the true camera system and the geometric approximation by the pinhole model, so-called distortion functions are added to the model. These distortion functions are defined as 2D functions in the image plane. In fact, they deflect the vision rays but do not prevent their passage through the projection center. They may have a continuous functional description or a discrete representation, according to each pixel. The most common distortion functions are radially symmetric and decentering distortion.

Distortion compensation is key to achieving very accurate results in 3D-reconstruction tasks. Hence, many papers (e.g., [16,17,18]) have been published concerning this task. However, some authors have described the effect of changing distortion depending on the distance between camera and depicted object, e.g., Magill [19], Brown [20], Fraser and Shortis [21] and Shortis et al. [22].

A variety of camera-calibration techniques, including distortion compensation using PM, have been developed in the past. Substantial contributions have been given by Brown [20], Tsai [23], Heikkilä and Silven [24], and Zhang [25], among others. Remondino and Fraser [26] compared several calibration strategies according to achievable accuracy.

The pinhole model has many advantages in 3D reconstruction by triangulation, e.g.:

- Their proximity to physical reality;
- Epipolar geometry [27] can be used for finding point correspondences;
- Ease of implementation;
- Software tools for calibration and 3D calculation commercially available.

Common software tools for pinhole-camera calibration include BINGO [28] and other software tools [29].

#### 3.1.2. Ray-Based-Camera Model

**Figure 2:**
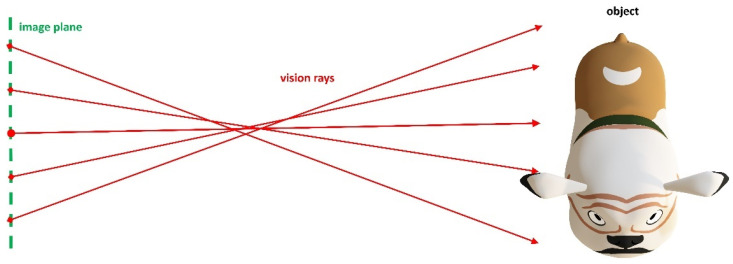
Ray-based-camera model. Rays do not necessarily intersect.

The ray-based or generic camera model (RM) assigns every camera pixel a ray in the scene (see Figure 2). Hence, it can be used universally without systematic measurement errors [30]. However, it is only very rarely used for 3D-reconstruction tasks. Some of the reasons for this are:

- Accuracy improvement is typically marginal;
- Classical epipolar geometry cannot be used to find point correspondences;
- Calibration process is very onerous, and there is no standard calibration methodology;
- Commercial software tools for calibration and 3D calculation are not available; hence, own implementation is necessary.

Despite these disadvantages, some authors, such as Wong et al. [14] have presented approaches to the usage of the ray-based-model for 3D reconstruction tasks.

In this work, some approaches to overcoming these disadvantages are given, namely:

- Presentation of a suitable calibration strategy;
- Realization of technical implementation of the PM and 3D-point-calculation procedure;
- Significant systematic-error reduction by a factor of four.

Additionally, some examples of test-specimen measurements are documented.

### 3.2. The Duality of Ray-Based and Pinhole-Camera Modeling

In this section, we show that the pinhole model can be easily transformed into the ray-based model and that the ray-based model can even be represented by the pinhole model with some small additions.

#### 3.2.1. Representation Possibilities of the Ray-Based-Camera Model

In the ray-based camera model, every camera pixel is assigned to the ray in the scene which is mapped onto it. Hence, the representation of the model requires a 3D ray representation to every pixel. One feasible representation is a 3D point and a 3D direction vector for every camera pixel. There are five parameters per pixel if the direction vector is normalized. Hence, a complete camera representation can be achieved using a suitable storage of five-element real number vectors. A suitable software implementation can be easily achieved.

**Figure 3:**
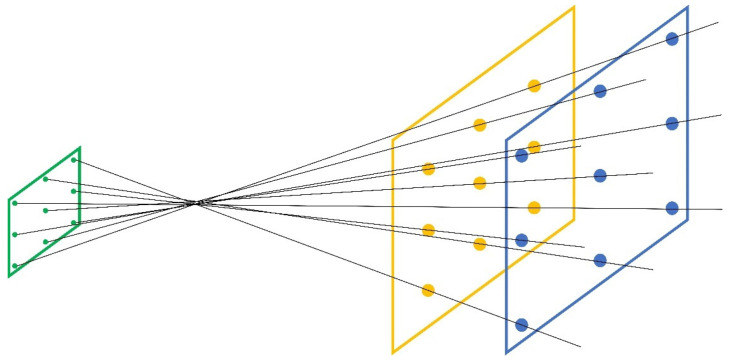
Image plane (green) and two reference planes *E*_1_ and *E*_2_ in the WSC.

Another method to represent the RM is the fixation of two planes *E*_1_ and *E*_2_ in the world co-ordinate system (WCS) and the intersection points of the pixel-corresponding rays with these two planes. For simplicity, it would be useful to take parallel planes, possibly also perpendicular to one of the co-ordinate axes of the WCS. For an illustration of this, see Figure 3. The two reference planes can also be used to characterize more than one camera through the RM representation.

Starting with these two planes in the WCS, a pinhole representation of the RM is also possible. Together with two distortion functions *f*_1_ and *f*_2_ corresponding to *E*_1_ and *E*_2_, which achieve the intersections of the planes *E*_1_ and *E*_2_ in the same points as the RM presentation, a third method of RM presentation is given.

#### 3.2.2. Translation of the Pinhole-Camera Model into the Ray-Based Model

With the input of the pinhole-calibration parameter set of a camera C_1_, including intrinsic and extrinsic parameters according to the WCS and a distortion function, a simple transformation into the RM can be obtained. This is achieved through selection of the 3D-pixel co-ordinates *P_i_* in the WCS as starting points of the rays *r_ij_* and calculation of the ray-direction vectors *v_i_* using the intersection of *P_i_* with *O* and, optionally, subsequent normalization of the direction vectors. The following equations express the calculation of the 3D points *q_ij_* of the RM and the ray normal vectors *v_ij_* by the set of PM-calibration parameters:

(1) $$\begin{array}{cc} q_{ij}= & R\times \left(p_{ij}+\Delta r_{ij}\right)+O\\ & v_{ij}=\left|p_{ij}-O\right| \end{array}$$

where:

- **R** is the extrinsic rotation 3 × 3 matrix of the camera in 3D world co-ordinates as part of the PM;
- *O* = (*x*_0_, *y*_0_, *z*_0_)*^T^* is the projection center in 3D world co-ordinates as part of the PM;
- *p_ij_* represents the metric positions of the camera pixels at index (i,j) in the intrinsic PM, z-component equals the negative value of the principal distance, and the origin (0,0) of x- and y-component is the principal point;
- Δ*r_ij_* represents the distortion vectors of the camera pixels at index (i,j), z-component equals 0, Δ*x*- and Δ*y*-components are derived from the distortion function of the PM;
- *q_ij_* represents the 3D starting points of the rays in the WCS corresponding to the pixels (i,j) as part of the RM;
- *v_ij_* represents the normalized 3D-ray vectors in the WCS corresponding to the pixels (i,j) as part of the RM.

#### 3.2.3. Translation of a Ray-Based Model into a Pinhole Model

In order to obtain a PM representation of a given RM, the following steps should be performed:

- Select two planes *E*_1_ and *E*_2_ in the WSC;
- Intersect the planes with all rays *r_i_* of the model and obtain points *e_i_*^1^ and *e_i_*^2^;
- Select the projection center *O*, principal distance *c*, and normal vector of the image plane of the PM;
- Intersect the image plane with the rays through *e_i_^j^* (*j* = 1, 2) and *O* and obtain points *p_i_*^1^ and *p_i_*^2^ in the image plane;
- Calculate the differences between *e_i_^j^* (*j* = 1, 2) and the image co-ordinates in the image planes of the corresponding pixels and obtain *d_i_^j^* = (*x*, *y*)*_i_^j^*, (*j* = 1, 2) as values of the distortion functions *f*_1_ and *f*_2_.

This algorithm translates any ray-based-camera model into a pinhole model with two distortion functions in the same WCS, together with two well-defined planes in the space. The resulting conversion corresponds to an extended pinhole model with an arbitrary distortion function, which can be approximated by the model of Grompone et al. [18], for example. Two pinhole models in the same WCS together with two planes define a ray-based model and vice versa.

### 3.3. Calibration Approaches

We call the refinement of the initially transformed model representation calibration of the ray-based model. Here, we suggest different approaches, one of which we eventually followed.

#### 3.3.1. Estimation of a Variable Principal Distance

This approach is suitable when we have a region in the measurement volume, where our pinhole calibration generates 3D-measurement results with low systematic error. Next, we can define a spatial plane in this region and replace the radially symmetric distortion function by a variable principal distance function (see [31]), including variable projection centers for each radial distance around the principal point. This new modeling can be transformed back into ray-based representation.

**Figure 4:**
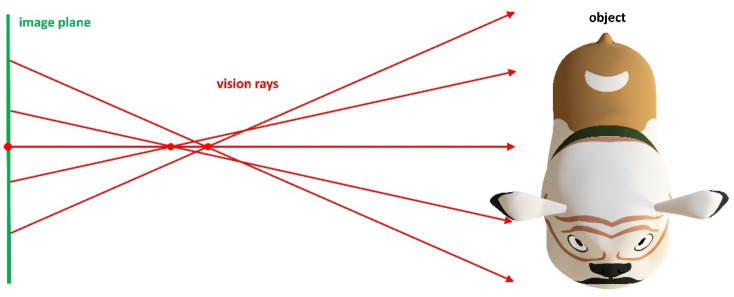
Variable principal distance. Rays corresponding to pixels with common distance to principal point intersect.

Figure 4 illustrates the idea of a variable principal distance instead of a radially symmetric distortion function.

#### 3.3.2. Connection of Two Pinhole Calibrations

This approach is suitable when we have one good calibration for close measurement objects and another for distant objects. We can merge these two calibrations by matching the extrinsic camera positions and place two spatial planes corresponding to the main calibration distances into the measurement volume. The intersection points of the camera rays with these two planes define the ray-based-camera model’s calibration.

Ideally, the same intrinsic and extrinsic parameters are enforced, but only the distortion functions are separately determined for the two distances.

#### 3.3.3. Calibration by Plane and Ball-Bar Measurements

**Figure 5:**
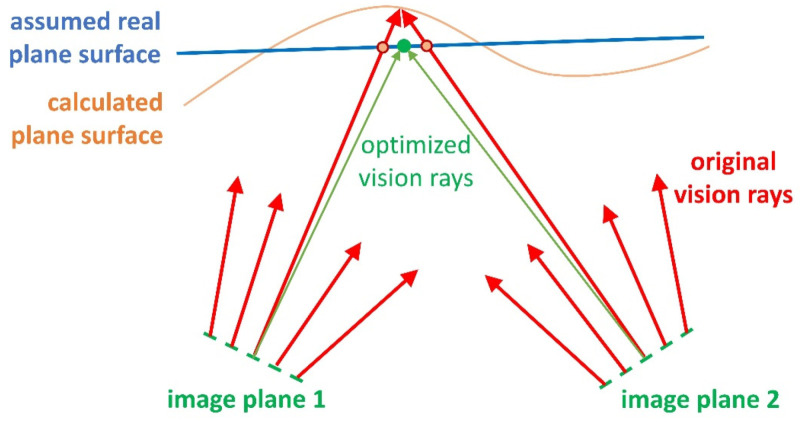
Enforcement of planarity by ray manipulation for one plane.

**Figure 6:**
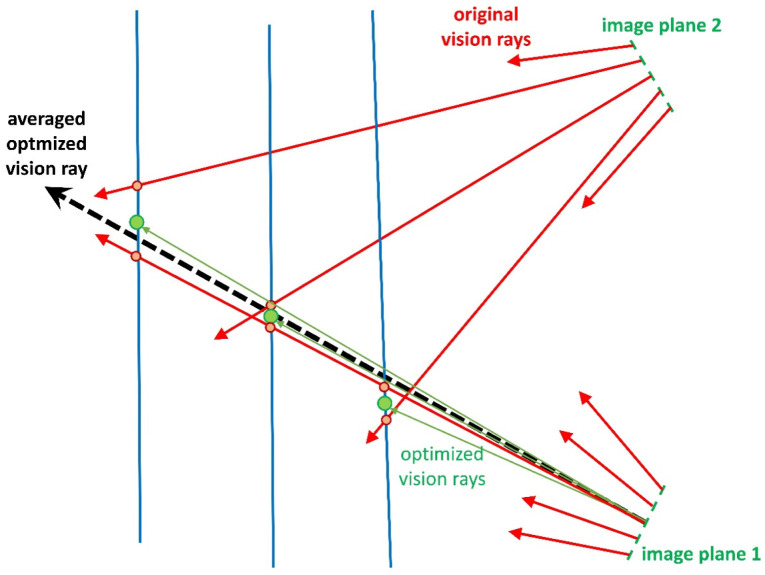
Different distances of ray manipulation corresponding to the same pixel of camera 1 leading to a weighted (dashed) ray.

This calibration approach can be used when we have a number of plane measurements at several distances from the cameras. We use the initial model (equivalent to the PM) and generate 3D point clouds of the plane measurements. We fit exact planes *E_i_* (see Figure 5) to these point clouds and enforce the position of the measured points on *E_i_* through symmetric manipulation of the two involved rays from both cameras. Note that one ray (of the master camera) is assigned to a pixel, and the other ray is an averaged ray, obtained by the rays of the four surrounding pixels. This process changes multiple times for almost all rays in the model. Each change is weighted, and an averaged ray is calculated for every pixel. This process should be performed iteratively until acceptable results are obtained. See Figure 6 for an illustration.

This strategy improves the rays according to errors in the measurement depth. However, lateral systematic errors are not necessarily corrected. Hence, additional analysis and, possibly, correction are necessary.

Analysis of a possible systematic lateral error can be detected by ball-bar measurements at different distances. When a functionally describable drift in the ball-bar length measurement is detected, this can be compensated by an appropriate manipulation of the rays, which does not influence the planarity of the measured planes.

The proposed iterative algorithm **AR** for the calibration of a stereo-camera arrangement represented by the ray-based model can be outlined as follows:

- Perform pinhole calibration;
- Transform the pinhole representation into the ray-based model;
- Perform measurements of a plane and a ball bar at different distances using the initial calibration;
- Start of iteration:
  ○ Fit planes to the point clouds;
  ○ Calculate mean distances of the points from the planes;
  ○ If mean distance is below a given threshold or maximal number of iterations is obtained, then proceed to end of iteration;
  ○ Enforce position of the measured points directly in the fitted plane and correction of the estimated systematic lateral error by symmetric manipulation of the rays in both cameras;
  ○ Weighted averaging of the manipulated rays;
- End of iteration.

A second method to obtain the RM is the omission of lateral-error compensation within the iteration process and subsequent correction. One procedure to achieve this is the following:

- Description of the systematic lateral error (radial or separated in X- and Y-direction) by a polynomial ***P*** (of very low degree) in the WCS;
- Transformation of all 3D points in the WCS by ***P***.

However, this compensation method would destroy the pure ray-based modeling of the cameras and finally increase the effort of the process of 3D data calculation.

Another suggestion is the subsequent manipulation of the model rays in order to compensate the systematic lateral errors.

In the next section, we give a proposal for our sensor system.

### 3.4. Sensor Setup and Specimen for Experiments and Measurement-Data Generation

#### 3.4.1. Laboratory Setup

**Figure 7:**
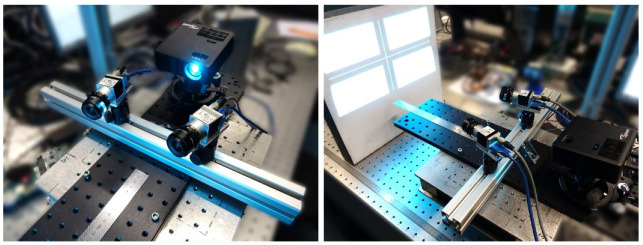
Laboratory setup with projector (left) and setup with plane stone slab (right).

In order to check the developed software modules and to perform the experiments described in the next sections, a laboratory setup consisting of two monochrome measurement cameras (Basler acA800-510um, Basler AG, Ahrensburg, Germany) with extreme-wide-angle lenses (Kowa LM5NCL, f = 4.5 mm, Kowa Optimed Deutschland GmbH, Düsseldorf, Germany) was used. For structured illumination generation a commercially available beamer (Optoma ML750e, Optoma, Taipei, Taiwan) was used. Figure 7 shows the setup. We used a small lens aperture (16.0) in order to obtain a large depth of focus within the measurement volume.

#### 3.4.2. Calibration Bodies and Test-Measurement Specimen

**Figure 8:**
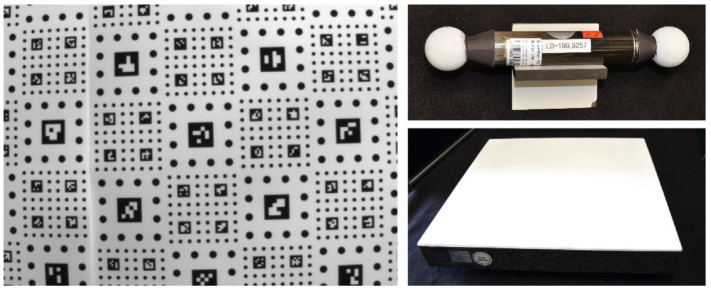
ArUco marker board (left) as primary calibration tool, and ball bar (upper right) and plane stone slab (lower right) for calibration refinement according to the ray-based model.

For calibration according to the pinhole model, ArUco markers [32] were used (see Figure 7). Additional specimens for evaluation measurements were ball bars with calibrated length between the sphere center-points and a stone slab with calibrated planarity (Figure 8 right). The ball bars were manufactured by the Metronom AG company, an enterprise that no longer exists. The stone slab was manufactured by the Planolith company (Planolith GmbH, Aschaffenburg, Germany) [33]. Maximal height deviation was 4.3 µm, according to the calibration certificate. The ball bars and stone slab were also used for the refinement of the calibration in the ray-based representation.

### 3.5. Generation of Evaluation Data

Using the laboratory setup, a number of datasets for plane and ball-bar measurements were generated. All specimens were recorded at several distances from the sensor setup covering tall possible measurement volumes. In practice, distances were limited to between 200 mm and 900 mm.

## 4. Experiments and Results

### 4.1. Implemention of the Ray-Based Model

#### 4.1.1. Data Structure for the Ray-Based Camera Model

For the data structure for the ray-based camera model, a file structure was selected. Every ray corresponding to one camera pixel can be described by a real number vector with six or five components: the starting-point co-ordinates *p* = (*x*,*y*,*z*) and the ray-direction vector *v_dir_* = (*dx*,*dy*,*dz*) in the WCS. Since vector *v_dir_* can be normalized to length one, *dz* can be omitted.

#### 4.1.2. Interpolation of Rays

Assume a pair of corresponding pixels *p*_1_ and *p*_2_ in both cameras. Typically, the pixel co-ordinate of *p*_1_ in the first (master) camera corresponds to a sub-pixel co-ordinate of *p*_2_ in the second (slave) camera. Let *p*_2_ have the sub-pixel indices *x* and *y* with *x* = *i* + *k* and *y* = *j* + *l* with integers *i* and *j* and rational numbers *x*, *y*, *k*, and *l* with 0 ≤ *k* < 1 and 0 ≤ *l* < 1. Next, we consider the surrounding pixels and corresponding rays *r_ij_*. Because of the discrete ray storage corresponding to the camera pixels, an interpolation of the rays for sub-pixel co-ordinates is necessary, similar to common pixel interpolation. We suggest the usage of the sub-pixel co-ordinates as the starting point of the interpolated ray *r_xy_* and a weighting of the normal vectors by bilinear interpolation. Alternatively, spline interpolation can be used. The interpolated vectors are calculated as follows:

(2) $$v_{ij}=w_{1}v_{ij}+w_{2}v_{ij+1}+w_{3}v_{i+1j}+w_{4}v_{i+1j+1}$$

where *w*_1_ = (1 − *k*) · (1 − *l*), *w*_2_ = (1 − *k*) · *l*, *w*_3_ = *k* · (1 − *l*) and *w*_4_ = *k* · *l*.

#### 4.1.3. D-point Calculation

The calculation of the 3D points *Q_i_* is obtained by ray intersection of *r*_1_ and *r*_2_ corresponding to the pixels *p*_1_ and *p*_2_, respectively, and is given by the equation:

(3) $$Q_{i}=\frac{1}{2}\left(q_{1}+q_{2}+s^{\prime }\times v_{1}+t^{\prime }\times v_{2}\right)$$

with the straight-line equations of the rays *r*_1_ and *r*_2_ given by *g*_1_ = *q*_1_ + *s* · *v*_1_ and *g*_2_ = *q*_2_ + *t* · *v*_2_ with a certain distance *D* between a 3D point on *g*_1_ and another 3D point on *g*_2_. Next, a linear optimization algorithm [34] is applied to find *s’* and *t’*, at which point *D* becomes minimal.

This kind of calculation is equivalent to the common calculation using the pinhole model.

#### 4.1.4. Software Evaluation and Comparison of Data-Processing Times

The first practical experiment was the transformation of the pinhole calibration into the ray-based calibration without a change in the parameters in order to detect possible errors in the software generation. This experiment was successful, i.e., equivalent 3D point clouds were obtained using the same input data. The processing times were 15 s vs. 0.5 s in the case of using two MPix cameras in the stereo setup. For practical applications, the processing time of the RM is too long. However, software optimization should be possible, which should, in turn, make the usage of the ray-based modeling more efficient.

### 4.2. Calibration-Refinement Results

In the first step, the recorded data of the plane stone slab and the ball bar at measurement distances 200 mm, 300 mm, 400 mm, 500 mm, 600 mm, and 700 mm were used for refinement. The orientation of the slab was mainly perpendicular to the sensor’s main axis. The ball bar was primarily horizontally oriented. Only weak changes in the incidence angles occurred.

The manipulation of the rays was performed according to the algorithm described in Section 3.3.3. Both cameras were alternately used as master camera and slave camera. In every case, twelve iterations were performed.

The plane and ball-bar measurement data for the distances 250 mm, 350 mm, 450 mm, 550 mm, 650 mm, and 800 mm were used for the evaluation. According to flatness error *F_e_* and length measurement error *L_e_*, the results of the plane measurements are documented in Table 1. The flatness deviation is defined as the Euclidean difference of the maximum 3D value to the minimum 3D value of the measured points, according to a fitted plane. The noise was removed by the subtraction of six times the standard deviation from the difference value due to the 99.9% confidence interval and the assumption of a normal distribution of the random 3D error. For further details concerning *F_e_*, see [15] and [35].

**Figure 9:**
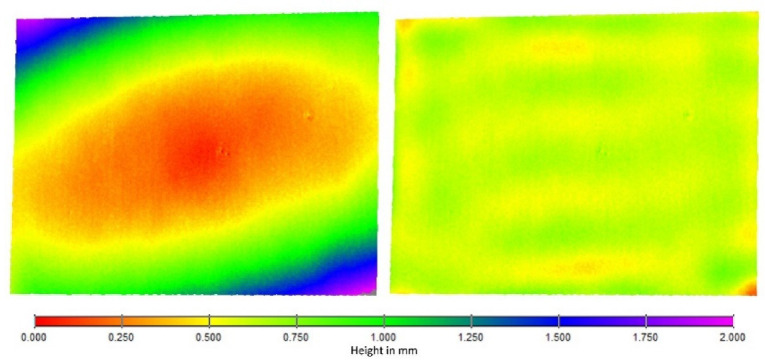
Example of a plane measurement at distance of 400 mm. False color representation with initial (left) and refined (right) calibration.

**Figure 10:**
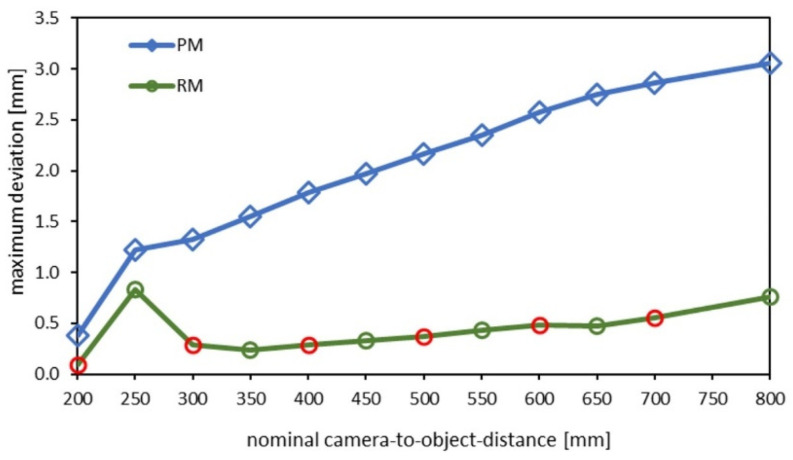
Flatness-deviation results. RM = ray-based calibration (green curve), PM = pinhole calibration (blue curve). Red circles mark the values used for refinement.

The results show a strong improvement in the planarity after the calibration refinement. An example of the 3D point cloud according to a fitted plane is shown in Figure 9. Figure 10 shows the distance-dependent flatness-deviation results before and after the calibration refinement. The results of the ball-bar measurements after the calibration refinement are documented in Table 2.

**Figure 11:**
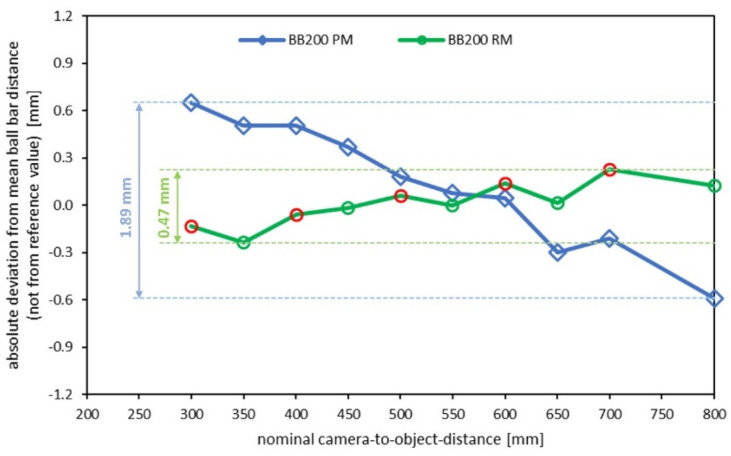
Length measurement results. RM = ray-based calibration (green curve), PM = pinhole calibration (blue curve). Red circles mark the values used for refinement.

The results show a significant effect. The systematic lateral error was significantly reduced. Figure 11 shows the distance-dependent length-measurement results of the ball bar before and after the calibration refinement.

## 5. Discussion

The presented results show the high potential of the suggested approach using the ray-based geometric modeling of cameras for photogrammetric 3D-reconstruction tasks. Additionally, the first measurement examples confirm the suitability of the suggested calibration methodology. However, there is still ample opportunity for improvements regarding both the measurement accuracy and the handling and performance.

The laboratory setup with extreme-wide-angle lenses was selected such that the measurement accuracy was initially very low in order to easily show improvements through the proposed methodology. In order to confirm the quality of the new technique, these improvements should also be verifiable using high-accuracy sensor systems with large measurement volumes and depths.

In order to achieve further improvements, the calibration-refinement procedure should be extended. The first necessary extension involves investigating whether the systematic lateral error of the initial pinhole calibration is radially symmetric or differs in the X- and Y-direction. This could be determined through additional ball-bar measurements with vertical alignment of the spheres.

The calculation time taken to obtain a 3D-reconstruction result for a scene was far too long for practical use. However, all the software implementations were realized with the goal of correctness and robustness and not of obtaining the shortest calculation time. Here, we see considerable potential for improvement.

Comparison with results previously published by other authors is difficult because 3D measurements of calibrated specimens, such as planes and ball bars, have not been published before. Li et al. [13] report an absolute maximal error of their projector system of 0.15 mm and a standard deviation of 0.02. However, this result cannot be compared to our measurements because the quantitative influence of the projector error on the 3D reconstruction result is not given. Other authors who introduced a ray-based model or calibration did not perform evaluable 3D measurements.

## 6. Conclusions and Outlook

In this work, a new concept for the ray-based modeling of cameras for photogrammetric 3D measurement was introduced.

The duality of the pinhole and ray-based-camera models was shown. That is, two pinhole models in the same WCS with distortion functions without restrictions together with two planes define a ray-based model and vice versa. This fact might be prospectively used for improvements to 3D-reconstruction tasks.

Possible applications with high potential for measurement-quality improvements are optical 3D sensors using wide-angle or fish-eye lenses, with large measurement depths or close object distances and using multi-camera systems with 3D data-fusion requirements. Additionally, optical 3D-measurement systems, in which vision rays pass through different optical media, e.g., for underwater use, are candidates for ray-based-model implementation.

A new approach to ray-based calibration based on ball-bar and plane measurements at different distances was given. Other calibration techniques are conceivable, e.g., two pinhole calibrations at different working distances in the same WCS.

The first results of 3D-measurement improvements to calibrated specimens (ball-bar and plane) are presented and discussed. Future work will address the following items:

- Performance and analysis of further experiments including vertical ball-bar alignments and slab orientations with multiple incidence angles with respect to the cameras;
- Performance of the calibration procedure using a setup with a higher basic accuracy;
- Implementation of the initial model transformation using the variable principal distance model;
- Extension of the epipolar geometry for ray-based-model representation for fast determination of point correspondences;
- Continuing refinement of the calibration procedure in order to obtain further improvements in the results;
- Optimization of the software implementation with the goal of the considerable acceleration of 3D calculation.

If a further improvement in the methodology can be achieved, the replacement of the pinhole camera model by the ray-based model seems possible for many applications. A considerable reduction in calculation time is necessary for acceptable software tools for 3D reconstruction tasks. Our goal is to apply the new modeling and methodology to the reduction in systematic 3D measurement errors when covering large measurement volumes with multiple stereo sensors.

## Figures and Tables

**Table 1:** Flatness error *F_e_* depending on measurement distance before and after calibration refinement. The measured reference area was about 300 mm × 200 mm.

Measurement Distance (m)	*F_e_* Original Calibration (mm)	*F_e_* Refined Calibration (mm)
0.20	0.38	0.07
0.25	1.23	0.55
0.30	1.32	0.10
0.35	1.55	0.10
0.40	1.79	0.14
0.45	1.97	0.15
0.50	2.16	0.18
0.55	2.35	0.21
0.60	2.58	0.23
0.65	2.75	0.30
0.70	2.87	0.36
0.80	3.06	0.53

**Table 2:** Length-measurement error *L_e_*, depending on measurement distances before and after calibration refinement. The reference length of the ball bar is 199.926 mm with 1.8 µm of uncertainty, according to the calibration certificate.

Measurement Distance (m)	*L_e_* Original Calibration (mm)	*L_e_* Refined Calibration (mm)
0.30	0.65	−0.13
0.35	0.51	−0.24
0.40	0.50	−0.06
0.45	0.37	−0.02
0.50	0.18	0.06
0.55	0.08	0.00
0.60	0.04	0.14
0.65	−0.30	0.02
0.70	−0.21	0.23
0.80	−0.59	0.13

## Data Availability

The data presented in this study are available on request from the corresponding author. The data are not publicly available due to a confidentiality agreement.
